# Differential effects of vision upon the accuracy and precision of vestibular‐evoked balance responses

**DOI:** 10.1113/JP275645

**Published:** 2018-04-16

**Authors:** Stuart W. Mackenzie, Raymond F. Reynolds

**Affiliations:** ^1^ School of Sport, Exercise, and Rehabilitation Sciences University of Birmingham UK

**Keywords:** balance, vestibular‐motor transformation, accuracy, precision

## Abstract

**Key points:**

- Effective balance control requires the transformation of vestibular signals from head‐ to foot‐centred coordinates in order to move the body in an appropriate direction.
- This transformation process has previously been studied by analysing the directional accuracy of the averaged sway response to multiple electrical vestibular stimuli (EVS).
- Here we studied trial‐by‐trial variability of EVS responses to measure any changes in directional precision which may be masked by the averaging process.
- We found that vision increased directional variability without influencing the mean sway direction, demonstrating that response accuracy and precision are dissociable.
- These results emphasise the importance of single trial analysis in determining the efficacy of vestibular control of balance.

**Abstract:**

Vestibular information must be transformed from head‐ to‐foot‐centred coordinates for balance control. This transformation process has previously been investigated using electrical vestibular stimulation (EVS), which evokes a sway response fixed in head coordinates. The craniocentric nature of the response has been demonstrated by analysing average responses to multiple stimuli. This approach misses any trial‐by‐trial variability which would reflect poor balance control. Here we performed single‐trial analysis to measure this directional variability (precision), and compared this to mean performance (accuracy). We determined the effect of vision upon both parameters. Standing volunteers adopted various head orientations (0, ±30 and ±60 deg yaw) while EVS‐evoked response direction was determined from ground reaction force vectors. As previously reported, mean force direction was orientated towards the anodal ear, and rotated in line with head yaw. Although vision caused a ∼50% reduction in response magnitude, it had no influence on the direction of the mean sway response, indicating that accuracy was unaffected. However, individual trial analysis revealed up to 30% increases in directional variability with the eyes open. This increase was inversely correlated with the size of the force response. The paradoxical observation that vision *reduces* the precision of the balance response may be explained by a multi‐sensory integration process. As additional veridical sensory information becomes available, this lessens the relative contribution of vestibular input, causing a simultaneous reduction in both the magnitude and the precision of the response to EVS. Our novel approach demonstrates the importance of single‐trial analysis in revealing the efficacy of vestibular reflexes.

## Key points

- Effective balance control requires the transformation of vestibular signals from head‐ to foot‐centred coordinates in order to move the body in an appropriate direction.
- This transformation process has previously been studied by analysing the directional accuracy of the averaged sway response to multiple electrical vestibular stimuli (EVS).
- Here we studied trial‐by‐trial variability of EVS responses to measure any changes in directional precision which may be masked by the averaging process.
- We found that vision increased directional variability without influencing the mean sway direction, demonstrating that response accuracy and precision are dissociable.
- These results emphasise the importance of single trial analysis in determining the efficacy of vestibular control of balance.

## Introduction

Because the vestibular system is locked within the skull, the signals it provides must be transformed from head‐ to foot‐centred coordinates for balance control (Lund & Broberg, 1983; Hlavacka & Njiokiktjien, 1985; Pastor *et al*. 1993; Fitzpatrick & Day, 2004; Mian & Day, 2009). For example, when leftward head motion is detected while facing forwards, a compensatory body movement to the right would be the appropriate response to maintain balance. However, if the head is turned 90 deg rightward, the same pattern of vestibular afferent feedback would require a backward body movement. This coordinate transformation process requires an accurate sense of head‐on‐feet proprioception (Dalton *et al*. 2017; Reynolds, 2017). Any breakdown in this process would compromise the efficacy of the vestibulo‐spinal reflex, which may increase fall risk.

This efficacy of the coordinate transformation process can be investigated using electrical vestibular stimulation (EVS) (Fitzpatrick & Day, 2004). EVS modulates activity of vestibular afferents, leading to a false sensation of body sway towards the cathode electrode. This evokes a compensatory sway response towards the anodal ear. This response is fixed in head coordinates, such that turning the head in yaw produces an equal rotation of the evoked sway direction. Previous studies have demonstrated the craniocentric nature of the EVS response by measuring the direction of the evoked body sway and/or ground reaction force vector at different head angles (Lund & Broberg, 1983; Mian & Day, 2009, 2014). Response direction is typically calculated by averaging sway responses to multiple EVS pulses of direct current, known as galvanic vestibular stimulation (GVS) (Inglis *et al*. 1995; Welgampola *et al*. 2013). More recently, the transformation process has been investigated using stochastic vestibular stimulation (SVS) (Dakin *et al*. 2007; Mian & Day, 2009). This involves application of a continuous randomly varying current lasting up to minutes. SVS offers advantages over GVS, including greater signal‐to‐noise ratio, and the ability to analyse the response in the frequency domain. GVS, by contrast, allows for the precise determination of response latency in the time domain (e.g. Nashner & Wolfson, 1974; Britton *et al*. 1993).

For both SVS and GVS, previous analysis has involved studying the conglomerate response to stimulation over time. For GVS, this consists of the average response to multiple stimuli. For SVS, cross‐correlations between stimulus and response time series are calculated for all possible directions over a prolonged period (≥30 s). The direction which produces the largest correlation value is then deemed to be the response direction. Both analysis techniques miss any transient or trial‐by‐trial variations in the direction of the sway response. These variations may be important for understanding the efficacy of balance control under more ethological circumstances. If we suffer a fall due to a transient error transforming vestibular input in motor output, an accurate *average* response is of little consolation. In other words, it is important to measure the precision, as well as the accuracy, of the vestibular‐evoked sway response.

Here we address this gap in the literature by measuring variability in the direction of the sway response to GVS and SVS. We ask two related questions. First, is the precision of the vestibular‐evoked sway response dissociable from its accuracy? Second, how are both parameters affected by vision? We hypothesise that closing the eyes will produce more variable (less precise) sway responses, while accuracy will be unaffected. Our rationale for this prediction is that the absence of vision will negatively affect head‐on‐body proprioception, and thus the ability to transform vestibular input into motor output for balance (Dalton *et al*. 2017; Reynolds, 2017). In fact, our results refute this hypothesis. Closing the eyes produced *less* variable responses. This occurred for both GVS and SVS, but was more clearly demonstrated using the latter technique. We discuss this unexpected finding in the context of a multisensory integration process. Accuracy, however, was unaffected by vision, confirming that precision and accuracy are indeed dissociable.

## Methods

### Ethical approval

The experiment was approved by the local ethical review committee at the University of Birmingham, and was performed in accordance with the *Declaration of Helsinki*, except for registration in a database. Informed written consent to participate was obtained from all participants.

### Participants

Twelve participants (nine males) aged 20–30 years (mean ± SD; 25 ± 2 years) with no known neurological or vestibular disorder were recruited.

### Protocol

**Figure 1:**
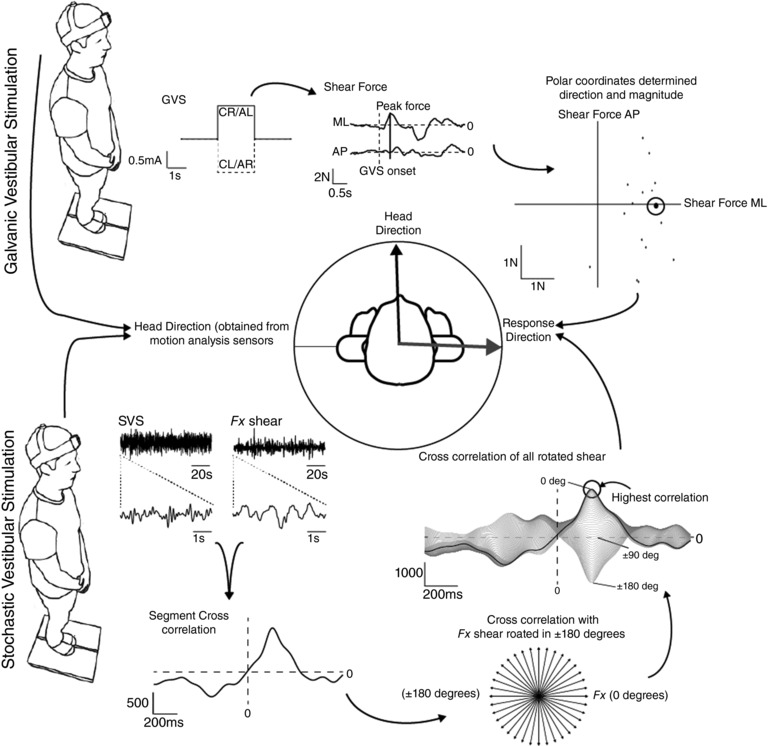
Analysis of EVS‐evoked postural responses (Top) GVS was delivered in a binaural bipolar configuration (1 mA, 1 s), evoking a reflex sway response that was recorded via a force platform in the form of ground reaction forces. Anode‐left data were inverted before combining with anode‐right trials. The timing of the peak force vector was first calculated from the averaged forces. Individual trials were then analysed by measuring the direction of the force vector within 200 ms of this time point. (Bottom) For SVS, SVS–force cross‐correlations were calculated for force vectors directed along all angles of a circle. The largest cross‐correlation determined response direction. A Polhemus motion tracker provided head orientation.

Participants stood in the centre of a force plate, unshod, with feet together and hands held relaxed in front of them for the duration of each 100 s stimulation period (Fig. 1). Prior to each trial participants were instructed to face one of five visual targets (±60, ±30 and 0 deg) located at eye level. This could be achieved through a combination of neck and trunk rotation until a head‐mounted laser crosshair became aligned with the target 1 m away.

EVS was delivered using carbon rubber electrodes (46 × 37 mm) in a bipolar binaural configuration. Two electrodes were coated in conductive gel and secured to the mastoid processes using adhesive tape. Stimuli were delivered from an isolated constant‐current stimulator (model 2200; AM Systems, Carlsberg, WA, USA). Two types of EVS were used: GVS and SVS. GVS was applied in sequences of 20 1 s impulses of 1 mA, separated by a 4 s gap. Positive values of current signify an anode‐right configuration. Each SVS period consisted of a 100 s stimulus. The stimulus waveform was generated by passing white noise through a low‐pass filter (5 Hz; 6th order Butterworth) and then scaling to give a root mean square value of 0.6 mA, and a peak amplitude of ±2 mA.

Each target angle (−60, −30, 0, +30 and +60 deg) and stimulation condition (GVS and SVS) was performed separately with eyes open and closed, giving a total of 20 conditions. Trial order was randomised and participants were allowed seated rest in between trials.

### Data acquisition

Head orientation was sampled at 50 Hz in the form of Euler angles using a Fastrak sensor attached to welding helmet frame (Polhemus Inc., Colchester, VT, USA). Sensor yaw was used to calculate head direction (i.e. rotation about the vertical axis). Any offset in yaw or roll angle between head orientation and sensor orientation was measured using a second sensor attached to a stereotactic frame, and subsequently subtracted. A slight head up pitch position was maintained throughout each trail to ensure that Reid's plane (line between inferior orbit and external auditory meatus) was horizontal, thus optimising the response to the virtual signal of roll evoked by vestibular stimulation (Fitzpatrick & Day, 2004). The evoked sway response was recorded in the form of ground reaction forces at 1 kHz using a Kistler 9281B force platform (Kistler Instrumente AG, Winterthur, Switzerland).

### Data analysis

#### GVS analysis

Analysis of GVS‐evoked shear force is depicted in the top half of Fig. 1. For each trial, any offset at stimulus onset was first removed from both mediolateral (*F*_*x*_) and anteroposterior (*F*_*y*_) force. Prior to individual trial analysis, we first averaged *F_x_* and *F_y_* traces across all trials within each condition. The time of the peak average force vector was then measured, and a window of ±200 ms either side of this time point was subsequently used to analyse each individual trial. The magnitude and direction (atan *F_x_*/*F_y_*) of the peak force vector within this time window was measured separately for all trials. This resulted in 20 individual trial directions for each condition, from which we could calculate the mean direction (i.e. accuracy) and its variance (i.e. precision) using circular statistics (see below). Response direction was referenced to head orientation, as measured by the Fastrak sensor.

After inverting anode‐left trials, there was no significant effect of polarity upon response magnitude (mean ± ST; AL 1.65 ± 1.01, AR 1.62 ± 1.02, *T*_89_ = 0.39, *P* = 0.70) or direction (*F*_1,178_ = 0.92, *P* > 0.34). Hence, both polarities were combined.

#### SVS analysis

Analysis of SVS‐evoked shear force is depicted in the bottom half of Fig. 1. We used a modified version of the technique described by Mian & Day (2009) whereby the cross‐correlation between the SVS stimulus and shear force is calculated. The component of the force vector is first determined for each degree of a circle (±180) to produce 360 separate force traces F_*ROTθ*_, using the following formula:

$$F_{\mathrm{ROT}\theta }\;\left(s\right)=\;F_{X}\left(s\right)\;\cdot \;\cos\theta +\;F_{Y}\left(s\right)\;\cdot \;\sin\theta$$

where s is sample. The SVS–force cross‐correlation is then calculated for each trace, and the angle which results in the largest cross‐correlation value is deemed to be the response direction. Initially we performed this analysis using the entire 100 s stimulation period. This was used to calculate the timing of the peak cross‐correlation response. To study response variance, we then split the data into segments and performed the same analysis again, determining peak correlation values at the time point derived from the full 100 s. We experimented with segments of differing lengths (1, 5, 10 and 20 s) and settled upon 5 s because it offered the greatest potential for detecting changes in variance between conditions (see Fig. 9 in Results). As for the GVS analysis, response direction was referenced to head orientation.

To determine response magnitude for SVS data, we measured the peak of the SVS–force cross‐correlation (units in mA·N), and normalised this by dividing it by the peak of the SVS–SVS autocorrelation (units in mA^2^). This resulted in a measure of gain that is independent of segment length (units in N mA^−1^).

### Circular statistical techniques

**Figure 2:**
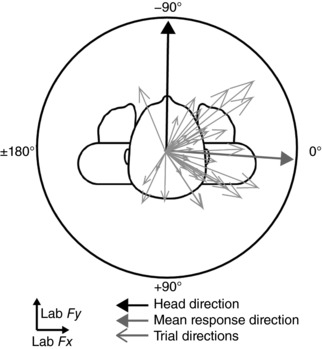
Individual trial analysis Mean head orientation and GVS‐evoked force vectors are shown by the solid black and grey arrows, respectively. Force vectors for individual trials are depicted by the thin grey arrows. These were used to calculate response precision, as measured by angular deviation.

For both GVS and SVS, response direction is represented by angular data. Therefore circular statistical techniques were implemented using the CircStat toolbox for Matlab (Berens, 2009). Angular conventions are represented in Fig. 2, which depicts a representative subject's responses to GVS during the head‐forward/eyes open condition.

To calculate mean directions, individual angles (α_1_, α_2_ …. α_n_) were first transformed to unit vectors in two dimensions (*r*_1_, *r*_2_ …. *r*_n_) by demanding that the circle had a radius of 1. Thus, the magnitudes of the individual subject responses did not affect the analysis of mean response direction. Rectangular coordinates of each unit vector were then calculated by applying trigonometric functions, where the sine and cosine of the angle give the *x*‐coordinate and *y*‐coordinate respectively:

$$\;r_{i}=\;\left(\begin{array}{c} cos\alpha_{i}\\ sin\alpha_{i} \end{array}\right)$$

Vectors (*r*_1_, *r*_2_, … *r_n_*) were then averaged to calculate the mean resultant vector (*r̅*):

$${}_{r}^{\_}\;=\;\;\frac{1}{N}\sum_{i}r_{i}$$

To compute the mean angular direction α̅, *r̅* is transformed using the four‐quadrant inverse tangent function. Angular deviation was calculated as a measure of response variance, as it is equivalent to the standard deviation in linear statistics (Batschelet, 1981) where *R* is the length of the mean resultant vector.

$$AD\;=\;\sqrt{-2(1-R)}$$

### Statistical analysis

A 2 × 5 repeated measures ANOVA (SPSS general linear model) was used to compare angular deviation and response magnitude across visual conditions and head orientations (visual condition: eyes open, eyes closed; head orientation: ±60, ±30, 0 deg). In all cases, where significant Mauchly's tests indicated violation of the assumption of equal variances, the degrees of freedom were corrected using the GreenHouse–Geisser technique. Response accuracy was determined by a linear fit between response direction and head direction.

We also performed correlations between response magnitude and variance. To do this, we determined response ‘error’ for each trial, measured as the angular difference between the individual trial direction and the mean direction. Pearson correlations were used to determine the significance of the magnitude–error relationship for each condition for each participant (see Fig. 8 below).

For all statistical tests, significance was set at *P* < 0.05. Mean angle and angular deviation/standard deviation [α̅ ± AD (STD)] are reported in the text and figures.

## Results

### Vestibular‐evoked sway responses

**Figure 3:**
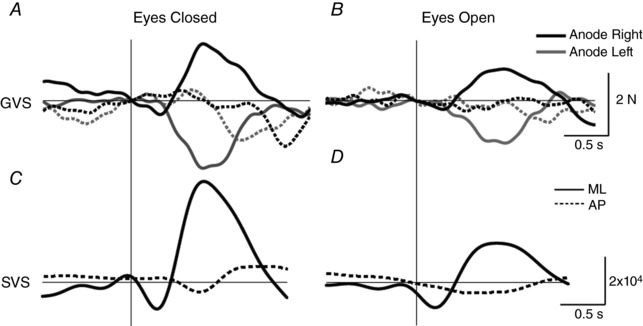
Representative EVS‐evoked forces with the head forward *A* and *B*, mean GVS‐evoked ground reaction forces for a representative subject. Mediolateral and anterioposterior forces are depicted by solid and dashed traces, respectively. *C* and *D*, SVS–force cross‐correlations for the same subject. Vertical lines depict time/lag zero for all traces. GVS stimuli started at time zero and lasted for 1 s.

Figure 3 depicts representative ground reaction force responses to vestibular stimulation in a subject standing with the head facing forwards. GVS evoked a polarity‐specific response, predominantly in the mediolateral direction (Fig. 3 *A* and *B*). SVS evoked a response in the same direction, as can be seen in the SVS–force cross‐correlation (Fig. 3 *C* and *D*). For both GVS and SVS, this subject's responses were larger with the eyes closed.

### Assessing response direction

**Figure 4:**
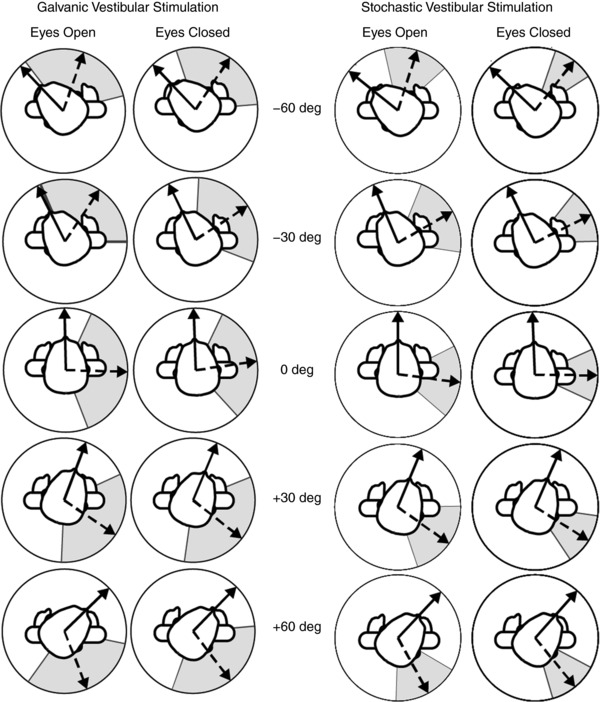
Mean and variance of evoked force vectors Group mean force vectors are shown separately for GVS and SVS. Mean head orientation and evoked force directions are shown by the solid and dashed black arrows, respectively. This response rotated in line with head orientation. The average of the within‐subject variability is represented by the grey shaded regions showing ± 1 angular deviation.

The effect of head orientation on the direction of the evoked force vector is depicted in Fig. 4. For all conditions, the mean force response (dashed line) is directed approximately 90 deg to head orientation (continuous line). As the head is turned between ±60 deg, the force vector turns by a similar amount for both GVS and SVS stimuli. The direction of the mean force vector was used to determine response accuracy. In contrast, response precision was determined by analysing the within‐subject variability of vector angles taken from individual trials/segments. This variability is depicted by the shaded areas in Fig. 4 which show angular deviation (circular equivalent of the standard deviation). For SVS, each 100 s stimulation period was split into 20 segments of 5 s.

### Response accuracy

**Figure 5:**
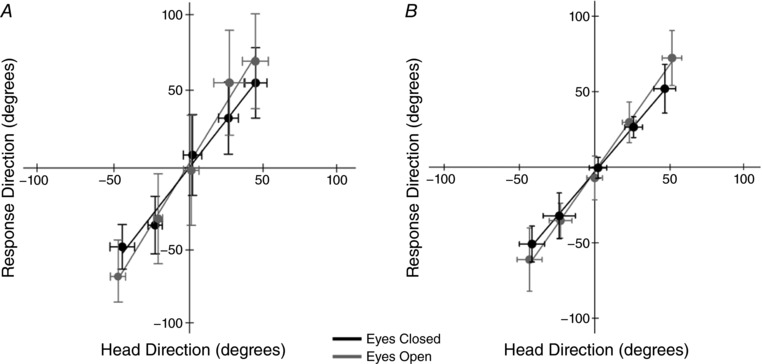
Response accuracy The effect of head orientation upon mean force vector direction is shown for GVS (*A*) and SVS (*B*). Error bars depict between‐subject standard deviation.

The effect of head orientation upon mean response direction is shown in further detail in Fig. 5. GVS‐evoked responses exhibited greater between‐subject variability than those produced by SVS stimuli (GVS; SD = 26.21. SVS; SD = 13.56). Furthermore, 3 of 12 subjects showed no significant correlation between head orientation and response direction for GVS stimuli (eyes closed: *R* ^2^ < 0.56; eyes open: *R* ^2^ < 0.48 *P* > 0.05). These subjects were removed from subsequent analysis and presentation of GVS responses (although their inclusion did not affect the outcome of any statistical analysis). In contrast, this relationship was significant for *all* subjects when using SVS stimuli (eyes closed: *R* ^2^ > 0.90; eyes open: *R* ^2^ > 0.85, *P* < 0.01). One subject was removed due to a malfunctioning of the Fastrak sensor system used to record head orientation.

For both GVS and SVS there was a significant linear relationship between head orientation and response direction (GVS *R* ^2^ = 0.88, *P* = 0.03; SVS *R* ^2^ = 0.95, *P* < 0.01). However, there was no effect of vision upon this relationship (ANOVA main effect of vision: GVS, *F*_1,8_ = 2.80, *P* = 0.13; SVS, *F*_1,10_ = 0.61, *P* = 0.45. *T* test on magnitude of regression slopes: GVS, *T*_8_ = 0.96, *P* = 0.364; SVS, *T*_10_ = −2.206, *P* = 0.07). This confirms that vision had no influence upon response accuracy, as measured by the direction of the mean force vector.

### Response precision

**Figure 6:**
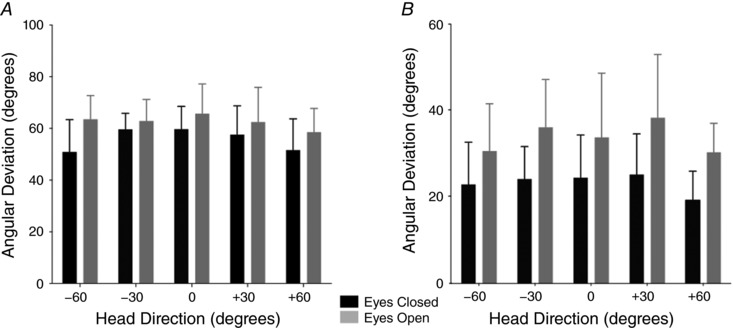
Response precision Within‐subject angular deviation is shown for GVS (*A*) and SVS (*B*), separately for all head orientations.

Individual trial/segment analysis was used to determine the variability of the evoked force vector (Fig. 6). There was a significant increase in angular deviation with the eyes open, both for GVS (11% increase, all head orientations combined; *F*_1,8_ = 15.16, *P* < 0.01) and for SVS (31% increase, all head orientations combined; *F*_1,10_ = 26.86, *P* < 0.01), indicating that vision actually *reduced* precision.

### Response magnitude

**Figure 7:**
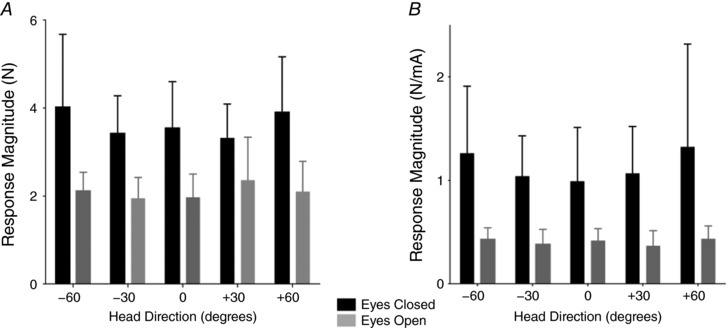
Response magnitude *A*, the magnitude of the GVS‐evoked force vector. *B*, stimulus‐response gain for SVS stimuli.

For GVS and SVS stimuli, response magnitude was determined by the peak force and the stimulus–response gain, respectively (Fig. 7). With the eyes closed, response magnitude was approximately doubled, both for GVS and for SVS (GVS, *F*_1,8_ = 65.74, *P* < 0.01; SVS, *F*_1,10_ = 30.32, *P* < 0.01). There was no effect of head orientation upon response magnitude (Fig. 7 *B*).

### Relationship between precision and magnitude

**Figure 8:**
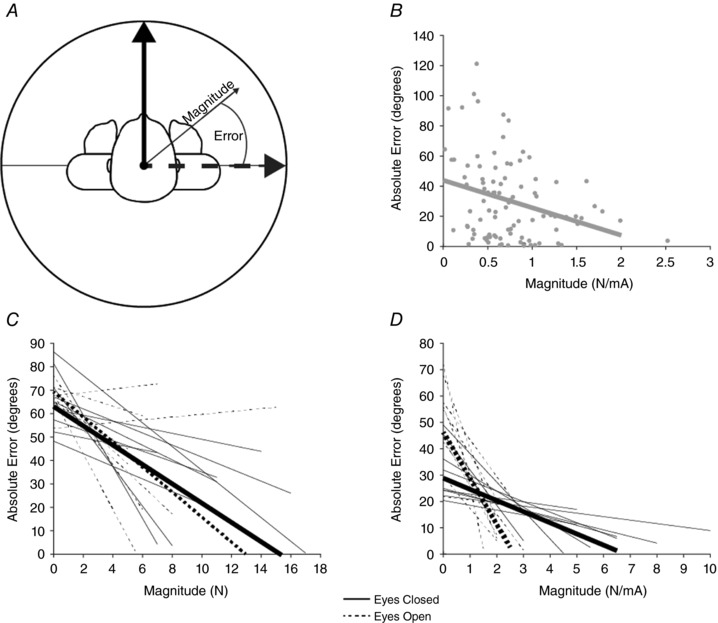
Relationship between response error and magnitude *A*, the absolute error between individual trial direction (thin grey arrow) and the mean response direction (dashed arrow) was calculated. The corresponding magnitude of each force vector for each trial was also recorded. *B*, a representative participant's SVS data and linear fit for an eyes open condition. *C* and *D*, regression lines for all subjects for GVS and SVS, respectively. Mean slopes and intercepts are represented by the thick lines.

To investigate the relationship between response precision and magnitude we calculated both the absolute error and the magnitude of each force vector for individual trials. Absolute error was calculated as the angular difference of individual force vectors from the mean vector, for each condition (Fig. 8 *A*). There was a tendency for larger responses to exhibit lower error (Fig. 8 *B*). This relationship was more consistent for the SVS response, where 9 of 11 participants exhibited a significant inverse correlation between these parameters, for both eyes‐open and eyes‐closed conditions (Fig. 8 *D*). For GVS, 4 of 9 participants produced a significant inverse correlation for both conditions (Fig 8 *C*).

### Effect of SVS segment length upon response precision

**Figure 9:**
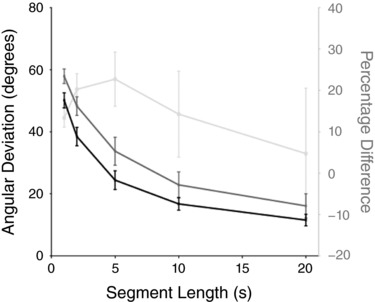
Effect of SVS segment length upon response variance Each 100 s period of SVS stimulation was split into segments of differing lengths, from 1 to 20 s. Eyes open and closed conditions are depicted by the solid grey and black lines. The percentage difference between visual conditions is shown by the feint grey line.

The analysis of SVS responses reported above was obtained by splitting each 100 s stimulation period into 20 5 s segments. Figure 9 shows the effect of altering segment length on directional variance for a forward facing orientation. Angular deviation systematically declines as segment length is increased. This may simply be due to the differing numbers of data samples produced by varying segment length. However, the values are consistently higher for the eyes‐open condition (F_4,44_ = 318, P < 0.01). The largest percentage difference between visual conditions occurred for the 5 s segment length (25% increase, mean ± SD eyes closed: 24.08 ± 9.53 deg, eyes open 34.67 ± 13.34 deg).

### Simulating changes in precision

**Figure 10:**
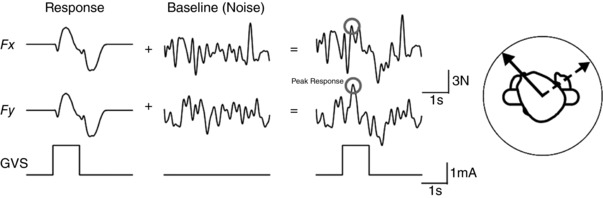
Simulating effects of response magnitude upon directional variance A GVS‐evoked force response was generated from averaged empirical data. This archetypal response was then summed with random noise to simulate baseline force variations. The peak response was used to calculate the direction of the resulting force vector for multiple artificial trials, allowing angular deviation to be calculated. Response magnitude and baseline noise were then independently varied to determine the effect upon angular deviation.

The above results suggest that vision increases the variability of the vestibular‐evoked balance response. However, there was an associated reduction in response magnitude with vision. It is therefore possible that change in variability is a direct consequence of this change in magnitude, rather than sensory reweighting for example (Fig. 8). To address this possibility, we generated artificial GVS responses where we could systematically modify response magnitude and observe the effect upon angular deviation (Fig. 10).

**Figure 11:**
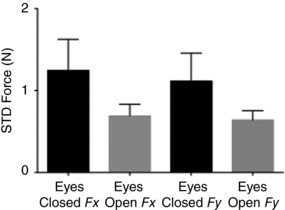
Baseline force variability Standard deviation of force data was calculated during a 1 s pre‐stimulus window for all GVS trials. There was a significant effect of vision upon baseline variability (*F*_1,7_ = 35.54, *P* = 0.001), but no effect of head angle or force direction (*F_x_* vs *F_y_*) (*P* ≥ 0.296).

Initial values of response magnitude and baseline noise were set to match the values observed empirically during the eyes‐closed GVS condition. We then decreased response magnitude by 42% to replicate the effect of opening the eyes. This caused a 39% increase in angular deviation, suggesting that the change in variance is indeed directly linked to response magnitude. However, this ignores variations in baseline force which might affect response variance. Analysis of the empirical data shows that baseline force variability decreases by 44% with the eyes open (Fig. 11). When we simulated this change alone (maintaining a fixed response magnitude), it caused a 27% *decrease* in angular deviation, opposing the effect of response magnitude.

**Figure 12:**
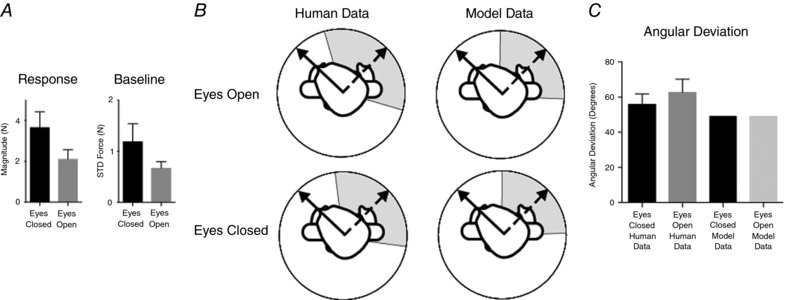
Comparison of empirical versus model data *A*, the empirically observed effects of vision upon response and baseline force magnitude were simultaneously implemented in the simulation. *B*, angular deviation was calculated for comparison against empirical data. *C*, there was minimal effect of these interventions upon the simulated angular deviation results. This contrasts with the 11% increase in angular deviation observed empirically when the eyes were opened.

When we simultaneously implemented the 42% decrease in response magnitude and the 44% increase in baseline force variability, the net effect was a 0.4% increase in response variability (Fig. 12). This compares to the empirically observed change of 11%. Hence, our simulation suggests that the observed changes in precision are not purely due to changes in response magnitude or baseline variability per se.

## Discussion

Our results confirm the craniocentric nature of the vestibular‐evoked sway response (Lund & Broberg, 1983; Hlavacka & Njiokiktjien, 1985; Pastor *et al*. 1993; Mian & Day, 2009). EVS stimuli evoked a ground reaction force directed towards the anodal ear, rotating in line with head orientation. The novel aspect of our study was to analyse the variability of this response in addition to its mean direction. When subjects opened their eyes, mean sway direction was unaffected. However, response variability increased, reflecting a reduction in precision. This demonstrates that the accuracy and precision of vestibular‐motor transformations for balance are dissociable. This raises the possibility that a person might exhibit poor balance control at any given instant, while appearing to sway accurately on average. The averaging process may therefore mask any deficits in vestibular control of balance.

We used two different methods of vestibular stimulation. The GVS stimulus consisted of a short‐lasting square‐wave pulse of direct current, allowing us to measure the direction of the vestibular response at a fixed instant in time. By measuring responses to multiple pulses, variability was readily ascertained. In contrast, SVS involved a continuous, long‐lasting and randomly varying current. To determine variability in this case, we quantified response direction over multiple segments of time ranging from 1 to 20 s, using the cross‐correlation method described by Mian & Day (2009). We settled upon a segment length of 5 s, because it showed the clearest distinction between visual conditions. Despite the difference in techniques, both GVS and SVS produced essentially the same result; vision had no influence upon the direction of the mean response, while variability *increased* with the eyes open. However, the practicality of both techniques differed. When using GVS, 3 of 12 subjects exhibited no clear relationship between head angle and response direction, and were thus excluded from further analysis. In contrast, this relationship was significant for *all* subjects when using SVS. Furthermore, the distinction between visual conditions was clearer in the SVS response, which exhibited a 31% increase in angular deviation with the eyes open, versus 11% for GVS. This is supported by previous work demonstrating greater signal‐to‐noise ratios for SVS‐evoked sway responses (Dakin *et al*. 2007; Reynolds, 2011). Of course, such differences may be partly attributable to the chosen stimulus parameters (Dakin *et al*. 2010). Varying the amplitude, number and frequency content of the stimulus current could conceivably alter angular deviation in ways we have not investigated here. Nevertheless, the qualitative similarity in results, regardless of the precise stimulus parameters, supports our assertion that vision increases the directional variability of the vestibular‐evoked sway response.

The observed effect of vision refutes our original hypothesis. We had reasoned that the sense of head‐on‐feet orientation would improve with vision. This would enhance the coordinate transformation of vestibular input into motor output for balance (Dalton *et al*. 2017; Reynolds, 2017). In contrast to our prediction, however, directional variability *increased* with the eyes open. How could vision reduce the precision of vestibular control of balance in this way? The answer to this apparent paradox may be sensory reweighting. We found that evoked force responses were ∼50% smaller with the eyes open. This concurs with previous findings showing that GVS‐evoked sway responses become smaller as additional veridical sensory information becomes available (Day *et al*. 2002). This has been demonstrated for tactile (Britton *et al*. 1993; Smith *et al*. 2017) and proprioceptive modalities (Day & Cole, 2002), as well as for vision (Day & Guerraz, 2007). The CNS must combine these sometimes divergent sources of information to compute a single estimate of the state of the body. This process has been likened to electoral proportional representation, with each sensory modality providing a vote towards the overall estimate of body orientation (Day *et al*. 2002). Hence, the relative contribution of any given modality will depend upon how much alternative sensory representation is available. The reduction in EVS‐evoked sway size with vision may therefore reflect down‐weighting of vestibular information. We also found a negative correlation between response magnitude and directional variability. We confirmed that this correlation was not due to inherent effects of noise in the force plate sensors (data not shown). Instead, it suggests that reduced precision is a direct consequence of the down‐weighting process. In other words, the CNS’ estimate of sway direction at any given time is less influenced by vestibular input. Hence there will be a greater influence of veridical visual cues upon sway direction.

Alternatively, it is possible that the changes in precision we observed are not directly attributable to sensory reweighting. The reduction in response magnitude could conceivably increase the variability of the sway force vectors via changes in signal‐to‐noise ratio. Specifically, a fixed level of random noise on the shear force signals (*F*_x_ and *F*_y_) would evoke greater angular changes for a smaller versus larger force vector. In this case, altered precision would not be caused by sensory reweighting per se. However, the results of our simple model suggest that this is not the case (Fig. 12). When we recreated the observed reduction in response magnitude, it did cause an increase in angular deviation. However, when we simultaneously implemented the empirically observed reduction in baseline force variability, angular deviation remained constant. This suggests that the effects of vision upon the precision of the vestibular‐evoked postural response are not mediated purely by changes in signal‐to‐noise ratio.

It is important to emphasise that the reduced directional precision that we observed with the eyes open does not reflect impaired balance control overall. Quite the opposite; in the absence of vestibular stimulation, baseline sway was 44% lower with the eyes open. Nevertheless, the analysis that we report here does offer a new method for analysing the efficacy of vestibular control of balance. Any increase in response variability in the absence of any other changes would indeed reflect impaired transformation of vestibular input. Furthermore, as our data demonstrates, it is possible for such changes to occur even when mean response direction remains accurate. This may be important for revealing potential contributions of vestibulo‐motor dysfunction towards increased fall risk, caused by age, sensory loss or neurological disease. Analysis of averaged responses may mask such deficits.

In summary, we observed a clear dissociation between the directional accuracy and precision of vestibular‐evoked balance responses. The directional variability of the EVS‐evoked sway response increased with the eyes open, while its mean direction was unaffected by vision. This paradoxical finding suggests that additional veridical sensory information leads to the down‐weighting of vestibular input for balance, resulting in an apparently less precise response.

## Additional information

### Competing interests

The authors have no conflicts of interest to declare.

### Author contribution

This study was performed at the school of Sport, Exercise and Rehabilitation Sciences, University of Birmingham, UK. SWM and RFR contributed to conception and design of the experiments, analysis and interpretation of data; drafting the article; and revising it for important intellectual content. SWM collected and assembled data. Both authors approved the final version of the manuscript.

### Funding

This work was supported by the UK Biotechnology and Biological Research Council (BB/P017185/1 & BB/I00579X/1). SWM is supported by an MRC‐ARUK PhD scholarship (MR/K00414X/1).
